# Supplementary figures and images for: Strain-specific genome evolution in Trypanosoma cruzi, the agent of Chagas disease
Source: PLoS Pathog. 2021 Jan 28;17(1):e1009254. doi: 10.1371/journal.ppat.1009254 (PMC7872254; doi:10.1371/journal.ppat.1009254)

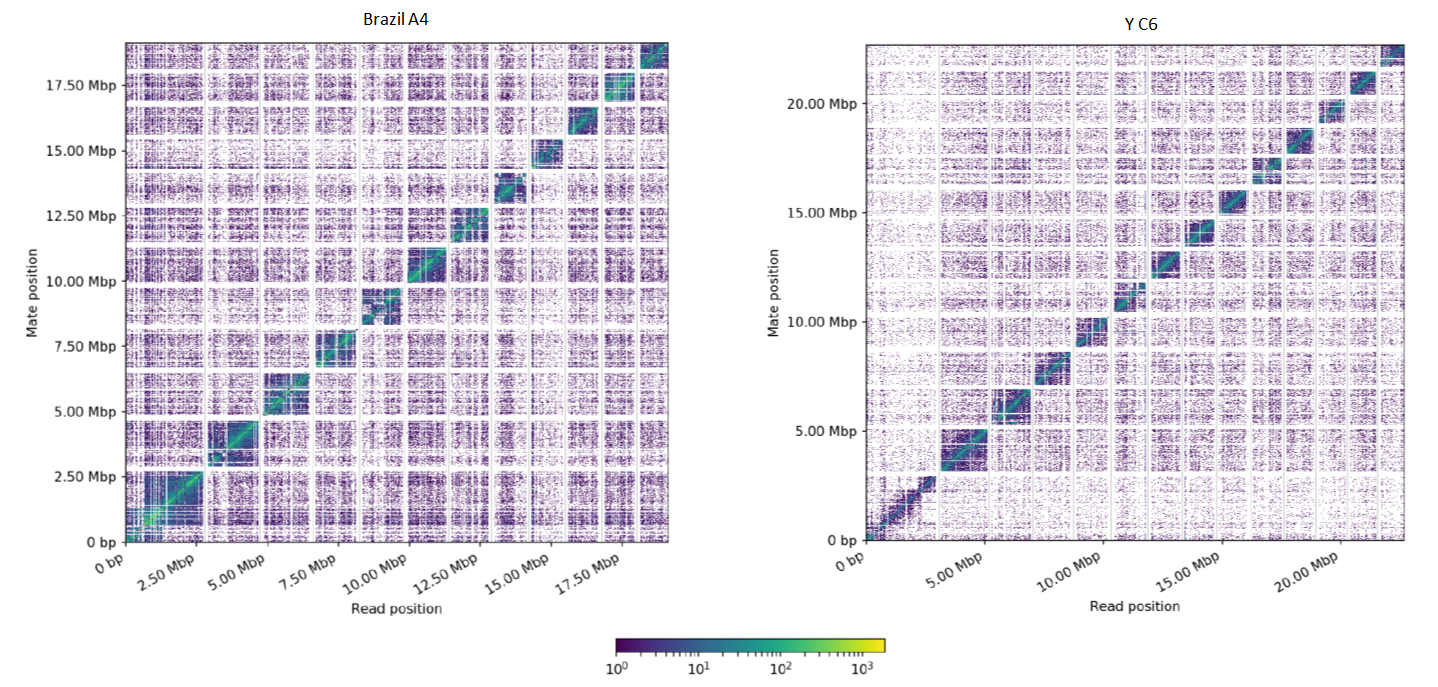

Supplement: S1 Fig — The x and y axes give the mapping positions of the first and second read in the read pair, respectively, grouped into bins. The color of each square gives the number of read pairs within that bin. White vertical and black horizontal lines have been added to show the borders between scaffolds. Only scaffolds > 1 Mb are shown. (TIF) [file ppat.1009254.s001.tif]

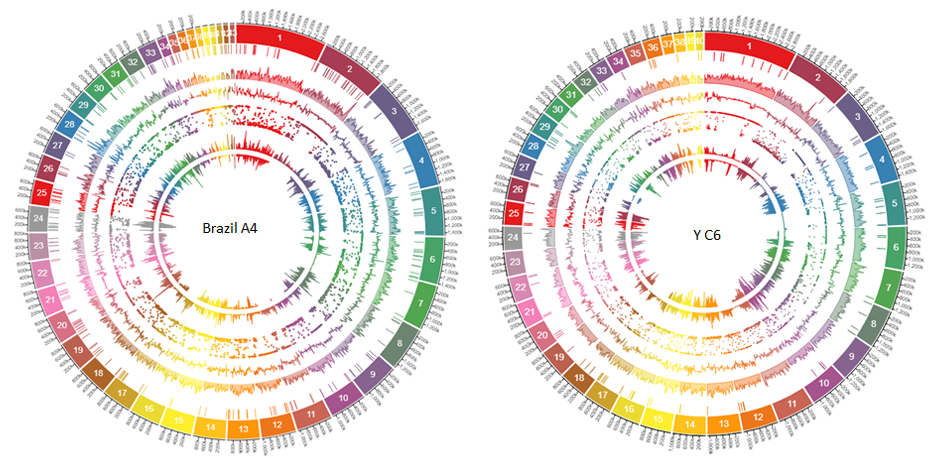

Supplement: S2 Fig — Tracks from outer to inner circles indicate: lengths, chromosome number, gaps, gene density (window size: 20kb, range: 6–23 in Brazil A4, 1–22 for Y C6), GC content (window size: 10kb, range: 0.36–0.70 in Brazil A4, 0.33–0.70 in Y C6), repetitive content (window size: 10kb, range: 0–10000), heterozygous SNPs (window size: 20kb, range: 0–120 in Brazil A4, 1–390 in Y C6) and heterozygous Indels (window size: 20kb, range: 65–1 in Brazil A4, 129–1 in Y C6). (TIF) [file ppat.1009254.s002.tif]

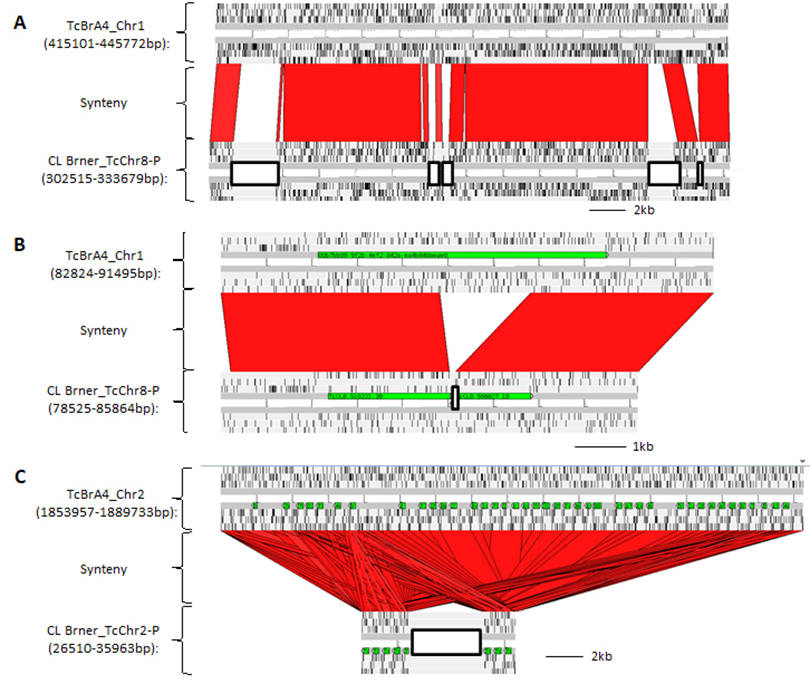

Supplement: S3 Fig — (A) An example of filled gaps. Syntenic regions between Chr1 in Brazil A4 and Chr8 in CL Brener were aligned with the Artemis Comparison Tool (ACT) [99]. All five gaps were filled in Brazil A4. (B) An example of recovered genes. Two pieces of an adenosine monophosphate (AMP) gene were identified flanking a gap, while the syntenic region in Brazil A4 shows the intact AMP gene. (C) An example of extended repeats. With 8 copies of histone H4 in Chr2 of CL Brener separated by a gap, the syntenic region of Brazil had the gap filled, extending the copy number of histone H4 to 41. Solid while boxes: gaps; green bars: genes. (TIF) [file ppat.1009254.s003.tif]

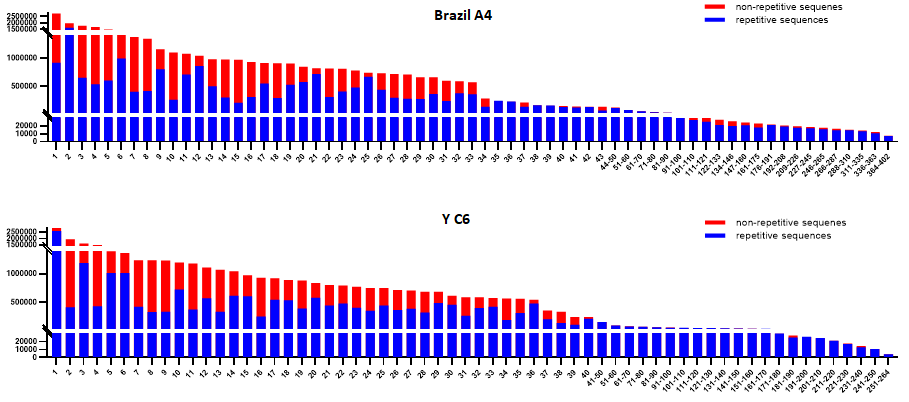

Supplement: S4 Fig — Chromosomes are calculated individually, while small scaffolds are calculated by averaging a range of scaffolds as indicated on the x axis. (TIF) [file ppat.1009254.s004.tif]

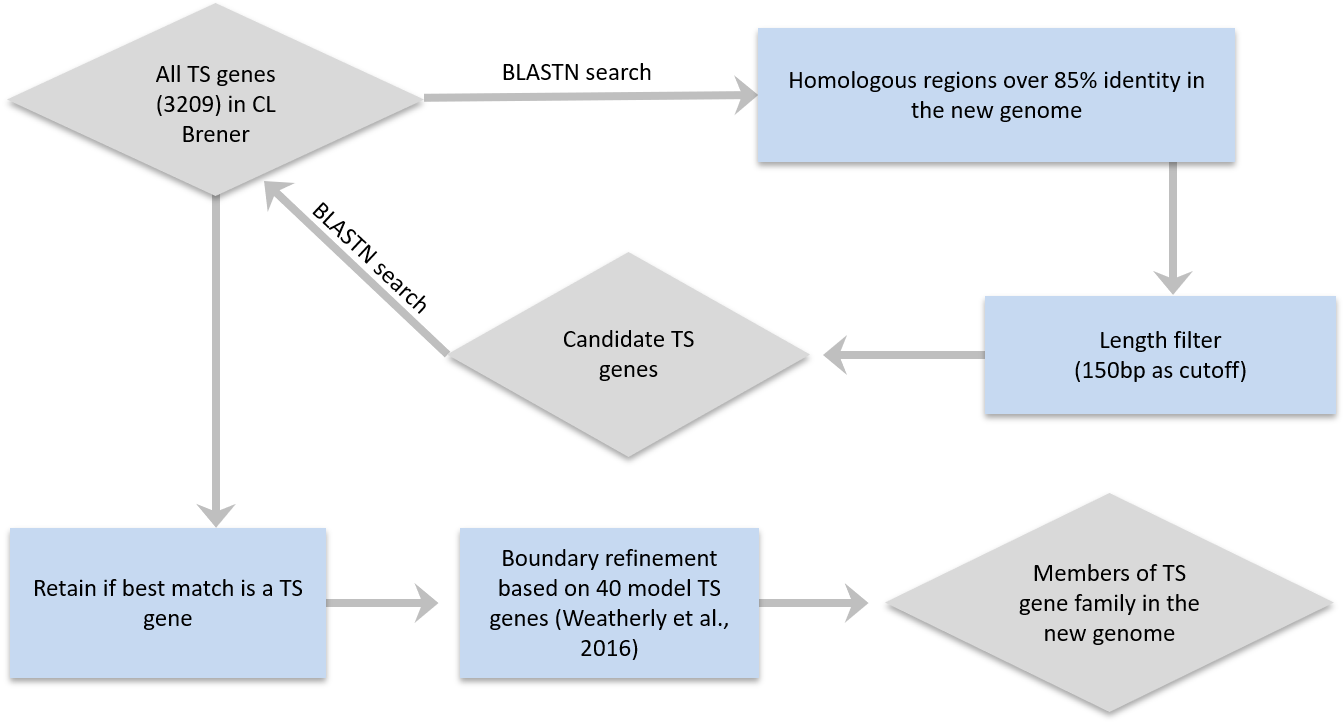

Supplement: S5 Fig — (TIF) [file ppat.1009254.s005.tif]

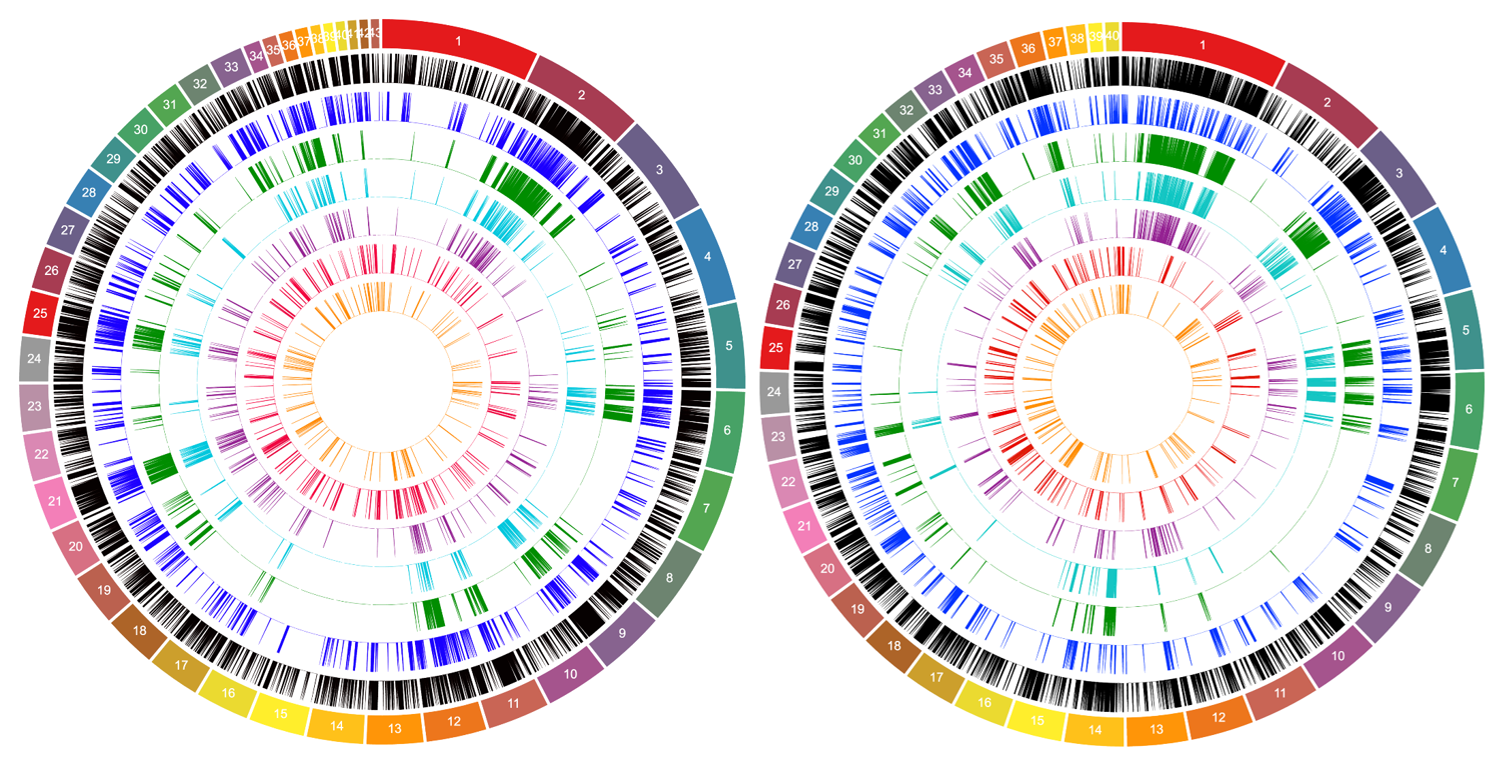

Supplement: S6 Fig — Rings from outer to inner: chromosome number, retrotransposons, TS, MASP, mucin, GP63, RHS and DGF-1 gene families. (TIF) [file ppat.1009254.s006.tif]

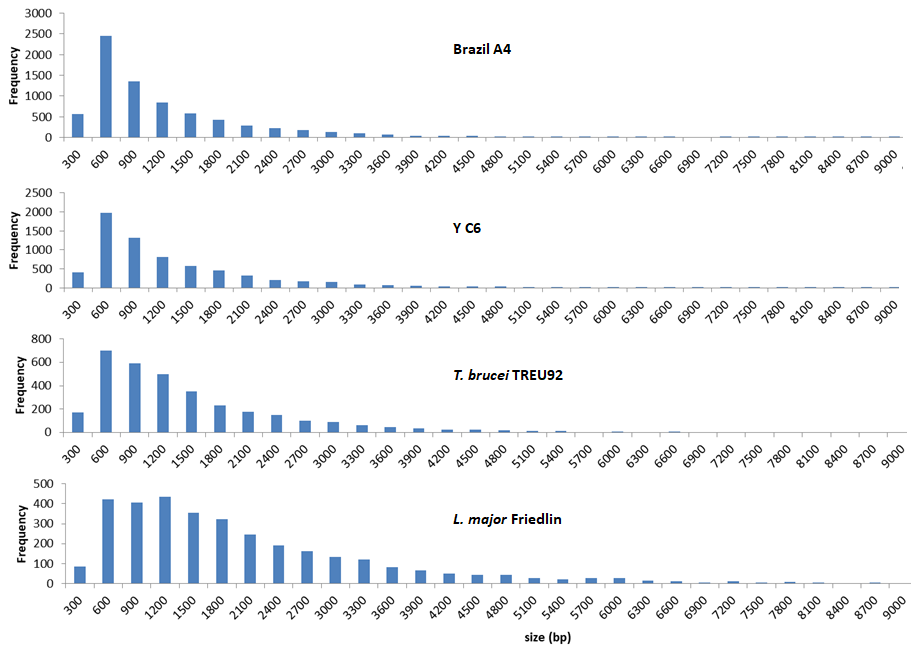

Supplement: S7 Fig — Genomes of T. brucei TRE92 and L. major Friedlin were downloaded from TriTrypDB database (https://tritrypdb.org/tritrypdb/) release-44 [100]. (TIF) [file ppat.1009254.s007.tif]

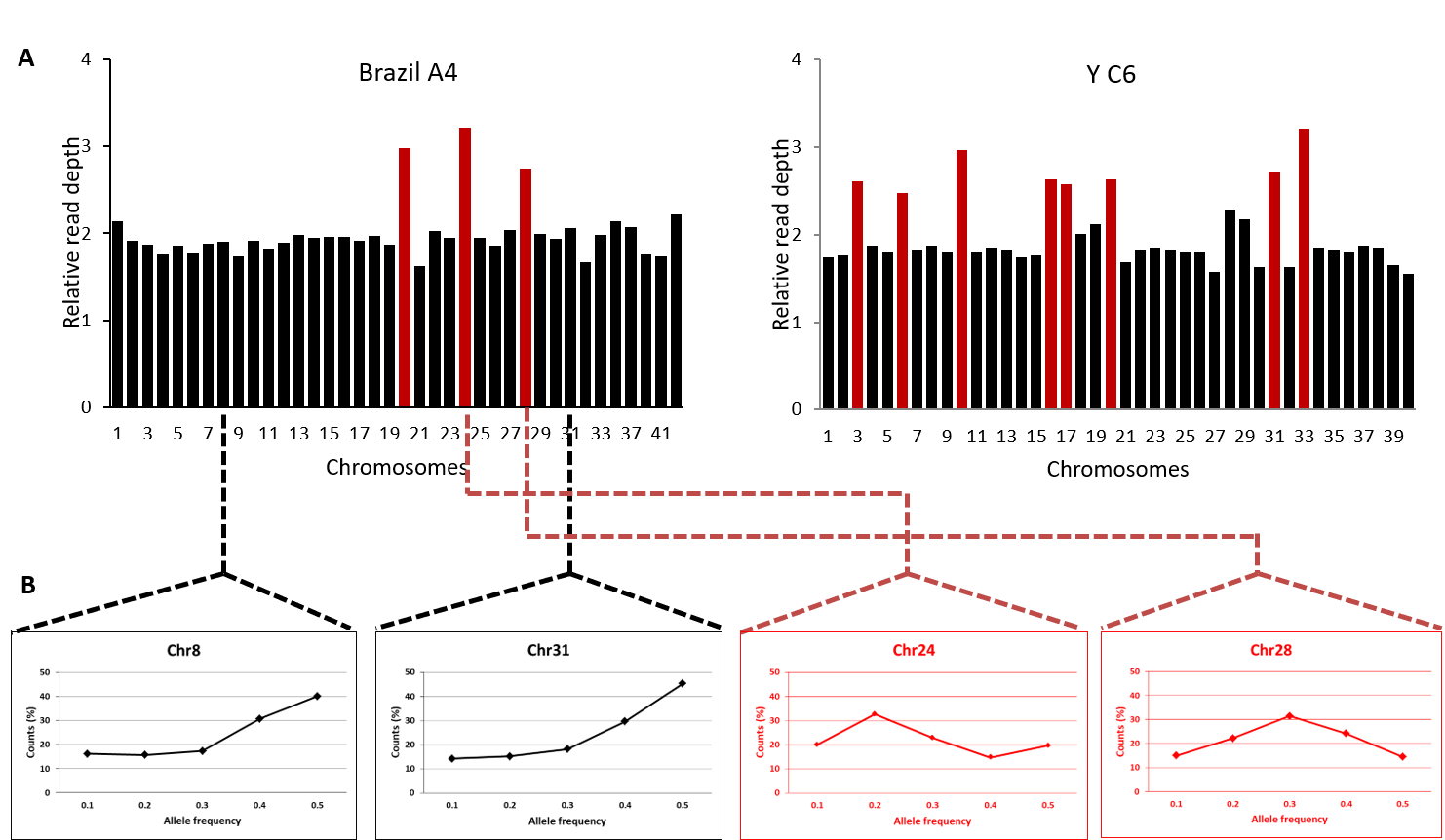

Supplement: S8 Fig — (A) Relative read depth of each chromosome normalized to the mean read depth of all chromosomes at non-repetitive regions. Chromosomes with more than two copies are indicated in red. (B) Allele frequency calculated by the proportion of heterozygous SNPs/Indels at the non-repetitive regions of each chromosome. ‘Counts (%)’ on the Y axis indicate the percentage of SNPs/Indels called at certain frequency which was calculated as previously described [39]. A diploid chromosome shows the peak of allele frequency around 50% as shown in Chr8 and Chr31, whereas an aneuploid chromosome shows peak of allele frequency lower than 50% as shown in Chr24 and Chr28 in Brazil A4. Note that 5 chromosomes (Chr35, 36, 38, 39 and 42) in Brazil A4 were not included in this analysis due to their high proportion of repetitive features. (TIF) [file ppat.1009254.s008.tif]

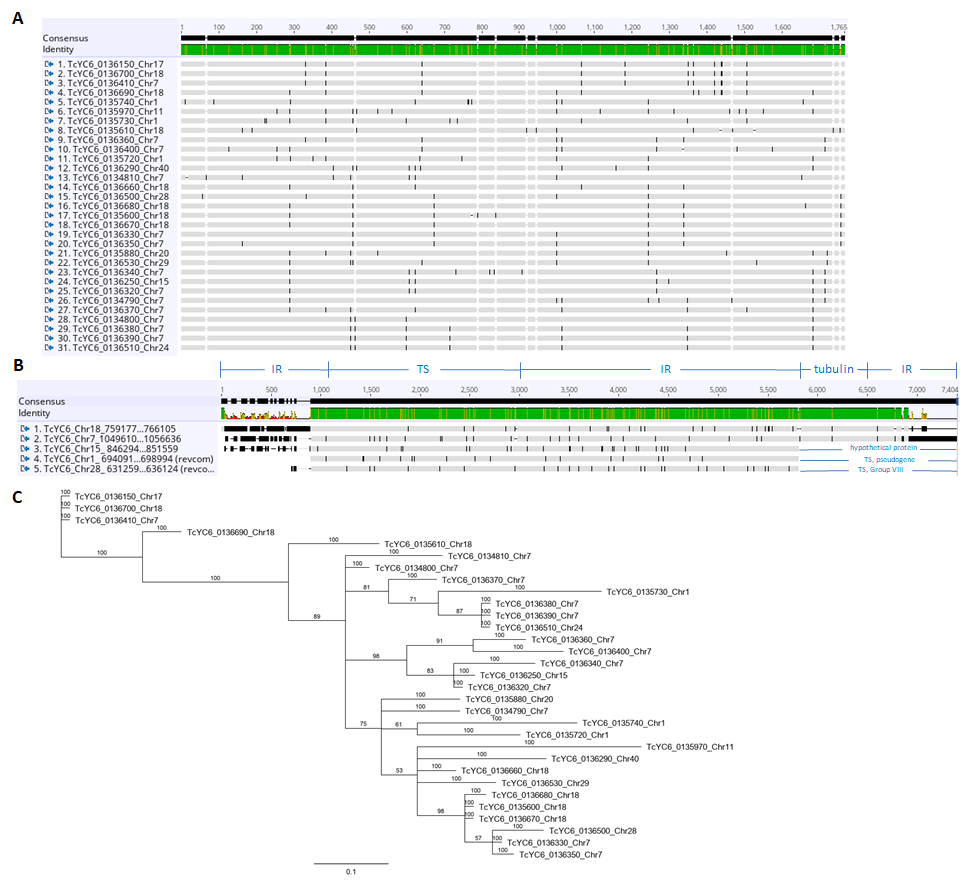

Supplement: S9 Fig — Alignment of TS (A) and their flanking regions (B) of the cluster in Fig 5, as well as the Bayesian inference of phylogeny of all the TS genes (C). IR: intergenic region. Alignments were analyzed using the same method as in Fig 4. (TIF) [file ppat.1009254.s009.tif]

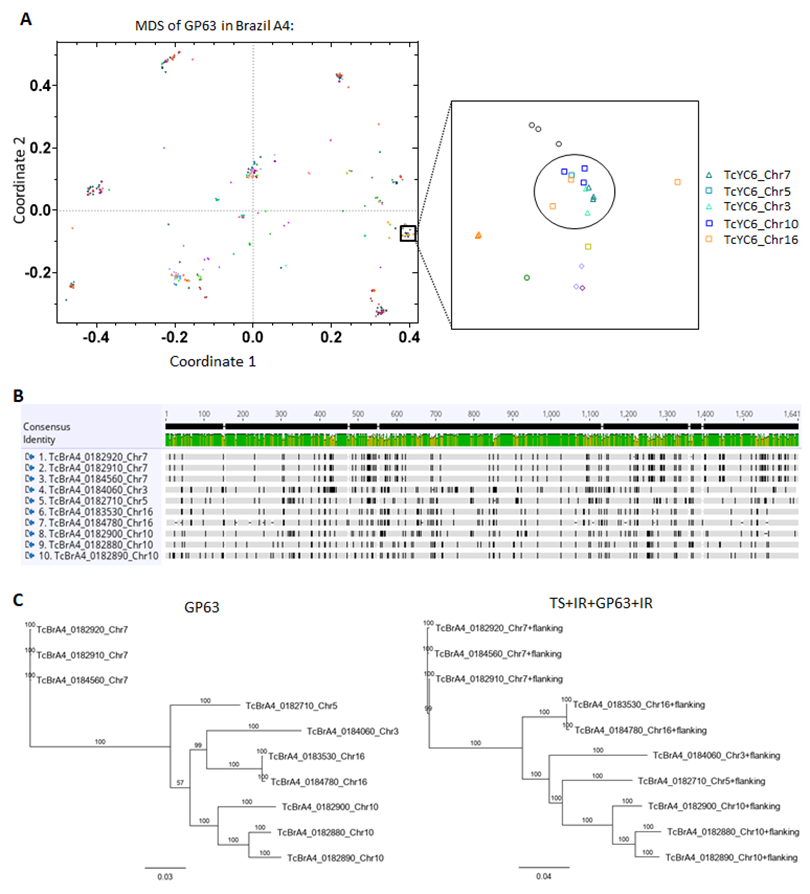

Supplement: S10 Fig — (A) A tight cluster of GP63 genes from MDS plot are distributed in different chromosomes. (B) Alignment of these GP63 genes showed high identity as well as a number of diversifications including SNPs and Indels. (C) Bayesian inference of phylogeny of GP63 in the cluster (left), and GP63 plus flanking sequences on both sides (right). IR: intergenic region. (TIF) [file ppat.1009254.s010.tif]

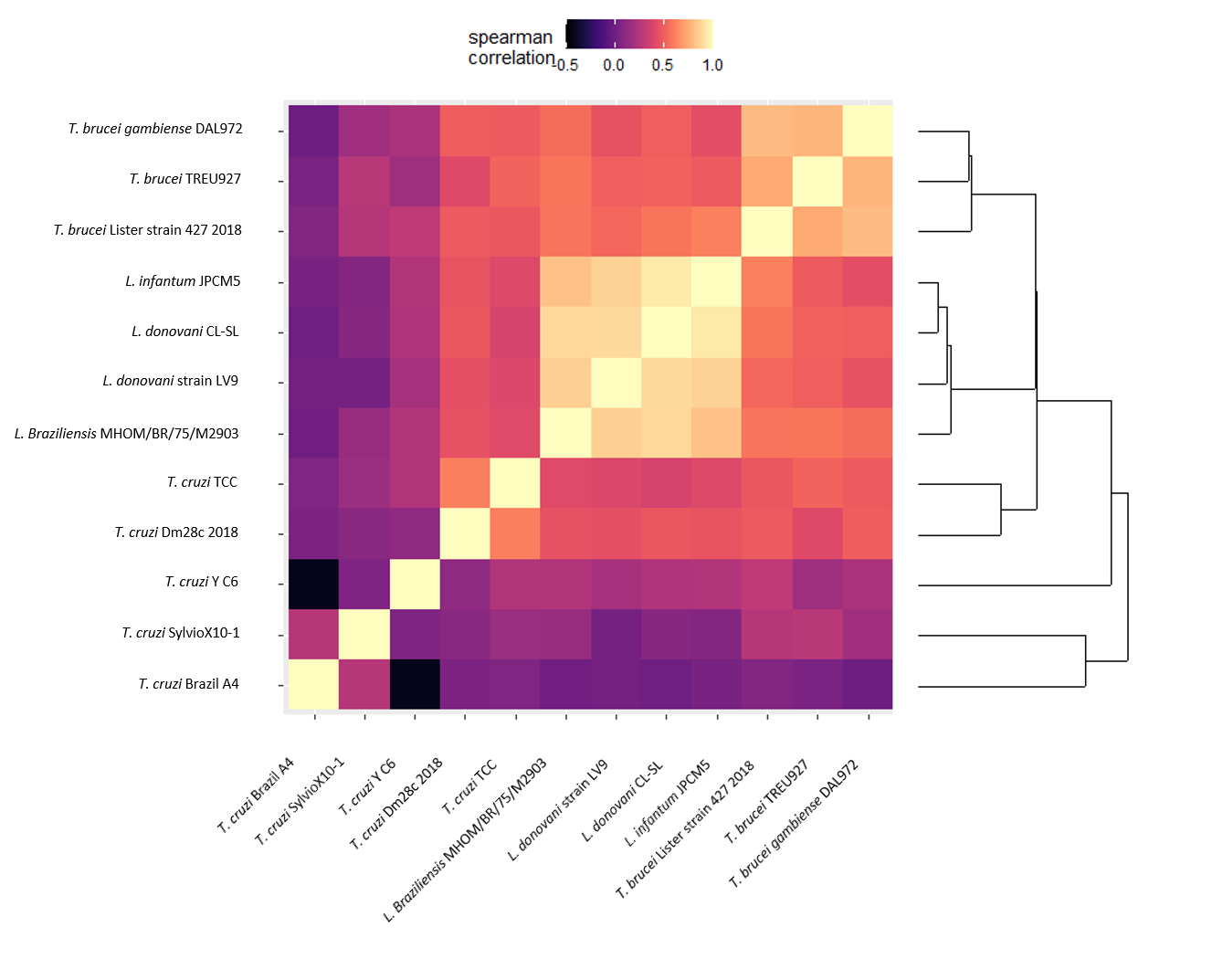

Supplement: S11 Fig — Copy numbers of 152 orthologous gene sets from S12 Table are highly correlated (Spearman correlation > 0.7) in pairwise comparisons between T. brucei strains and subspecies and between Leishmania species, but poorly correlated between T. cruzi strains (Spearman correlation in a range between 0.006 and 0.6). (TIF) [file ppat.1009254.s011.tif]

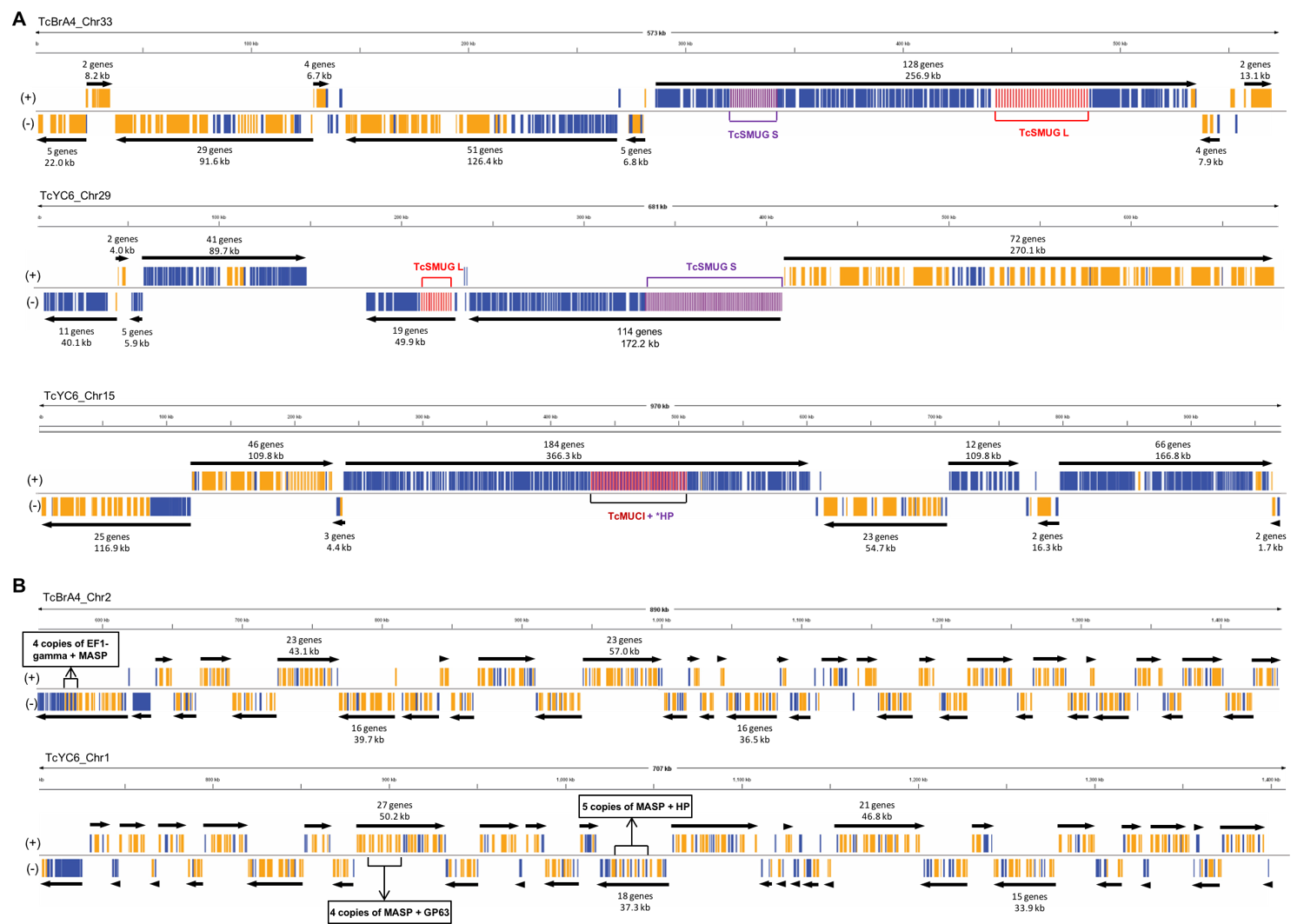

Supplement: S12 Fig — (A) Tandem arrays of conserved gene sets are contained within long PTUs devoid of large gene family members. Chromosomes containing TcSMUG S/L in Brazil A4 (top) and Y C6 (middle), and TcMUCI+*HP in Y C6 (bottom). In contrast, members of the large gene families, including some tandemly duplicated genes, show frequent strand switches and are in short PTUs (B). Blue bars indicate genes other than members of large gene families, while yellow bars indicate members of these gene families. (TIF) [file ppat.1009254.s012.tif]
