# Supplementary material for: Strain-specific genome evolution in Trypanosoma cruzi, the agent of Chagas disease
Source: PLoS Pathog. 2021 Jan 28;17(1):e1009254. doi: 10.1371/journal.ppat.1009254 (PMC7872254; doi:10.1371/journal.ppat.1009254)
Supplement: S1 Table — (PDF) [file ppat.1009254.s013.pdf]

S1 Table. Number of joins and breaks generated by Chicago or Hi-C libraries

	**Brazil A4**		**Y C6**	
	**Chicago**	**Hi-C**	**Chicago**	**Hi-C**
# of breaks	29	4	4	6
# of joins	170	135	42	64
