# Supplementary material for: Strain-specific genome evolution in Trypanosoma cruzi, the agent of Chagas disease
Source: PLoS Pathog. 2021 Jan 28;17(1):e1009254. doi: 10.1371/journal.ppat.1009254 (PMC7872254; doi:10.1371/journal.ppat.1009254)
Supplement: S2 Table — All sequences were retrieved from TriTrypDB database (https://tritrypdb.org/tritrypdb/) release-44. *No scaffolding was applied to these genomes, so no gaps were generated. **47 are not de novo assembled contigs or scaffolds, but rather pseudomolecules produced by aligning the core regions of scaffolds to the core regions of CL Brener reference genome. Therefore, although the genome showed higher N50 and lower L50, it left an extensively high number of gaps behind. ***Genome sequence is not available. (PDF) [file ppat.1009254.s014.pdf]

S2 Table. Summary of assembly metrics among all available *T. cruzi* genomes assembled by long-read sequencing

Genome	DTU	Method	Total size (Mbp)	Number of contigs or scaffolds	GC (%)	N50 (bp)	L50	Largest contig/scaffold length (bp)	# of gaps
Brazil A4	TcI	Illumina, PacBio, Chicago and Hi-C	45.56	402	51.58	914,771	17	2,738,928	295
Y C6	TcII	Illumina, PacBio, Chicago and Hi-C	47.22	262	51.58	889,019	18	2,951,016	106
Dm28c2018 [5]	TcI	Illumina and PacBio	53.27	636	51.56	317,638	47	1,645,565	0*
Sylvio X10/1 [7]	TcI	Illumina, PacBio and comparative genome	41.38	47**	51.57	1,006,492	14	3,116,433	1005
Berenice [8]***	TcII	Illumina and Nanopore	40.80	923	51.20	156,193	ND	926,516	0*
Bug2148 [6]	TcV	PacBio	55.16	929	51.27	200,364	64	1,305,792	0*
TCC [5]	TcVI	Illumina and PacBio	87.06	1236	51.72	264,196	92	1,305,230	0*

All sequences were retrieved from TriTrypDB database (https://tritrypdb.org/tritrypdb/) release-44.

*No scaffolding was applied to these genomes, so no gaps were generated.

**47 are not *de novo* assembled contigs or scaffolds, but rather pseudomolecules produced by aligning the core regions of scaffolds to the core regions of CL Brener reference genome. Therefore, although the genome showed higher N50 and lower L50, it left an extensively high number of gaps behind.

***Genome sequence is not available.
