# Supplementary material for: Strain-specific genome evolution in Trypanosoma cruzi, the agent of Chagas disease
Source: PLoS Pathog. 2021 Jan 28;17(1):e1009254. doi: 10.1371/journal.ppat.1009254 (PMC7872254; doi:10.1371/journal.ppat.1009254)
Supplement: S4 Table — 5 PacBio-assembled genomes with annotation were compared using either ‘eukaryota_odb9’ or ‘protists_ensembl’ datasets using default parameters. The ‘eukaryota_odb9’ dataset contains 303 single-copy genes conserved in 100 eukaryote species, while the “protists_ensembl’ dataset’ contains 215 single-copy genes that are present among 33 protist species. *Note that TCC is a hybrid strain, so its genome is a mixture of two haplotypes, while all other genomes contain one haplotype. (PDF) [file ppat.1009254.s016.pdf]

S4 Table. Assessment of genome assembly and annotation completeness using single-copy ortholog benchmarking

	Complete	Fragmented	Missing	Total
**Using eukaryota dataset**				
Brazil A4	224	19	60	303
Y C6	224	18	61	303
Dm28c2018	218	21	64	303
Sylvio X10/1	83	63	157	303
TCC*	224	18	61	303
**Using protists dataset**				
Brazil A4	119	0	96	215
Y C6	121	1	93	215
Dm28c2018	111	0	104	215
Sylvio X10/1	35	1	179	215
TCC*	124	0	91	215

5 PacBio-assembled genomes with annotation were compared using either ‘eukaryota_odb9’ or ‘protists_ensembl’ datasets using default parameters. The ‘eukaryota_odb9’ dataset contains 303 single-copy genes conserved in 100 eukaryote species, while the ‘‘protists_ensembl’ dataset’ contains 215 single-copy genes that are present among 33 protist species.

*Note that TCC is a hybrid strain, so its genome is a mixture of two haplotypes, while all other genomes contain one haplotype.
