# Supplementary material for: Strain-specific genome evolution in Trypanosoma cruzi, the agent of Chagas disease
Source: PLoS Pathog. 2021 Jan 28;17(1):e1009254. doi: 10.1371/journal.ppat.1009254 (PMC7872254; doi:10.1371/journal.ppat.1009254)
Supplement: S6 Table — (PDF) [file ppat.1009254.s018.pdf]

S6 Table. Copy number of large gene families characterized in the new genomes

	**TS**	**MASP**	**mucin**	**RHS**	**GP63**	**DGF-1**
Brazil A4	1647	1120	700	677	411	242
Y C6	1465	1061	797	796	427	265
