# Supplementary material for: Strain-specific genome evolution in Trypanosoma cruzi, the agent of Chagas disease
Source: PLoS Pathog. 2021 Jan 28;17(1):e1009254. doi: 10.1371/journal.ppat.1009254 (PMC7872254; doi:10.1371/journal.ppat.1009254)
Supplement: S7 Table — (PDF) [file ppat.1009254.s019.pdf]

S7 Table. Annotation summary

	Brazil A4	Y C6
**Protein-coding genes**		
Number of gene models	18,708	17,650
% of the genome	55.05	53.44
Mean CDS length (bp)	1,350	1,453
Gene density (genes per Mb)	407.75	372.31
GC (%)	53.66	54.13
% of hypothetical proteins	40.17	38.79
**Intergenic regions**		
Mean length (bp)	1,102	1,251
GC (%)	49.03	48.67
